# Renal coccidiosis in black skimmers in North America reveals an uncharacterized *Eimeria* lineage (Apicomplexa: Eimeriidae)

**DOI:** 10.1371/journal.pone.0345982

**Published:** 2026-04-16

**Authors:** Kátia R. Groch, Tiana L. Sanders, Elena M. I. Duran, Dallas Clontz, Jacquelyn K. Grace, Raquel R. Rech, Guilherme G. Verocai, Laura K. Bryan

**Affiliations:** 1 Department of Veterinary Pathobiology, College of Veterinary Medicine & Biomedical Science, Texas A&M University, College Station, Texas, United States of America; 2 Texas A&M Interdepartmental Program in Ecology and Evolutionary Biology, College Station, Texas, United States of America; 3 Veterinary Diagnostic Pathology, LLC, Polkton, North Carolina, United States of America; 4 Department of Ecology and Conservation Biology, Texas A&M University, College Station, Texas, United States of America; MARE – Marine and Environmental Sciences Centre, PORTUGAL

## Abstract

Black skimmers (*Rynchops niger niger*) are New World coastal seabirds that breed along the Gulf of Mexico, southern Atlantic and Pacific coasts of the United States. Black skimmer populations are declining in North America, and little is known about health and diseases of this species. An outbreak of mortality causing the death of approximately 160 juvenile black skimmers occurred in the West Galveston and East Matagorda Bay populations during the 2022 and 2023 breeding seasons. Necropsy, histopathology, and molecular genetic methods were utilized to investigate the cause of morbidity in six chicks that died or were euthanized. The main gross findings included bilateral renomegaly with multiple pale tan foci. Characteristic histologic findings were granulomatous nephritis and cloacitis with intralesional coccidia. Various developmental stages including immature gamonts, microgamonts, macrogamonts, and oocysts were present extracellularly and in the cytoplasm of epithelial cells of distal tubules, medullary collecting ducts, ureters, and cloacal epithelium. A few oocysts were in the cytoplasm of multinucleated giant cells. Additionally, schizonts were observed in the small intestine of one case. Genetic analysis of partial 18S rRNA gene revealed a previously uncharacterized *Eimeria* lineage affecting kidney and cloaca and another *Eimeria* lineage affecting the small intestine. To the best of our knowledge, this is the first report of *Eimeria* infection in the genus *Rynchops*. The significance of renal and intestinal coccidiosis in black skimmers is unknown; however, in the present work it appeared to cause morbidity and likely contributed to the recent mortality outbreak.

## Introduction

Coccidiosis is caused by intracellular protozoan parasites belonging to several genera of the family Eimeriidae within the phylum Apicomplexa. The genus *Eimeria* is one of the most well studied within this family, with over 1000 species identified infecting fishes, reptiles, birds, and mammals [1,2]. The eimeriid coccidia exhibit a complex, but direct life cycle comprising an exogenous stage and intracellular and extracellular stages with both asexual and sexual reproduction within the host. The transmission of coccidia occurs via ingestion of sporulated oocysts from the environment. In general, *Eimeria* species are host- and tissue-specific and commonly infect the intestinal tract or kidneys causing morbidity and increased mortality in birds. It has been suggested that in renal coccidiosis, sporozoites directly invade renal epithelial cells to initiate merogony followed by gametogony [3].

The pathology caused by coccidian infection varies with the species of *Eimeria*, host species, and site of infection [4]. Adults can be asymptomatic carriers and will shed oocysts, whereas the infection is usually severe or fatal in juvenile waterbirds, causing multiple large mortality events [3,5]. The disease primarily occurs in animals exposed to high infective doses or immunosuppressed [6], where it can cause multiple large mortality events [3,5], whereas low levels of infection may instead stimulate a protective host immune response [7]. Gross lesions are usually absent or include enlarged kidneys with white foci, and diagnosis is made through histopathology and molecular analyses [8]. *Eimeria* meronts and gamonts are predominantly found in the distal convoluted tubules and collecting ducts, causing inflammation, necrosis and tubular obstruction [9]. Renal coccidiosis has been reported in a variety of waterbirds, including cormorants, ducks, geese, swans, gulls, loons, puffins, and penguins [3,5,10–15].

The black skimmer (*Rynchops niger niger*; Linnaeus, 1758) is one of the three species of tern-like seabirds in the genus *Rynchops*, family Laridae (Charadriiformes). In the United States of America (USA), black skimmers breed on the southern California coast, Gulf of Mexico coasts and the Atlantic coast from Massachusetts to Florida. The most recent estimate of the population for the Atlantic and Gulf states of the USA is 30,000–35,000 pairs [16]. Though not listed as federally endangered, black skimmer populations have been declining for several decades [17–19]. Threats faced by black skimmers include degradation to nesting habitat, human disturbance, predation, sea-level rise and over wash, oil pollution, storms, and food availability [20,21]. Stressors such as these can directly cause mortality and can also increase the rate of infanticide, as observed in Florida [22], where infanticide was much higher in high stressed colonies than in relatively undisturbed colonies in New York and Texas [23,24].

Data pertaining to health and diseases of black skimmers are relatively limited despite their necessity for management and development of successful conservation plans. Prior research has shown that two mass mortality events in Florida were associated with systemic salmonellosis caused by *Salmonella enterica* serotype Typhimurium and severe arthritis and osteomyelitis caused by *Staphylococus aureus* infection secondary to penetrating sandspurs, respectively [25,26]. Additionally, variable burdens of ectoparasites (feather lice and mites) and endoparasites (intestinal cestodes or nematodes, and esophageal, intestinal and renal trematodes) are occasionally observed in black skimmers [26]. Lastly, *Plasmodium* sp. and *Haemoproteus* sp. were detected with 16% and 20% of prevalence, respectively, in black skimmers that reproduce and nest at Medium Solimões River region, Amazon Basin, in Brazil [27]. In Texas, important natural island breeding colonies of black skimmers in West Galveston and East Matagorda Bay have been monitored since 2018 by the Gulf Coast Bird Observatory to assess survival, movements, reproductive success and determine the causes of population decline. An outbreak of mortality of juvenile black skimmers occurred in the East Matagorda Bay population during the 2022 and 2023 breeding seasons and in the West Galveston population in the 2023 breeding season. In the present study we describe the pathologic findings and molecular analysis of renal coccidiosis in six black skimmers. To the best of our knowledge, this is the first report of *Eimeria* infection in a *Rynchops* species.

## Materials and methods

### Study area

Old Gulf Cut in East Matagorda Bay (28°43’ N, 95°49’ W) is enclosed by the Matagorda Peninsula and the tidal flats at the mouth of the Colorado River of Matagorda County on the Texas Gulf Coast. Gangs Bayou in West Galveston Bay (29°15’ N; 94°57’ W) is a long inlet in Galveston and Brazoria Counties, west of Galveston Island. Areas used regularly by skimmer colonies for nesting on Old Gulf Cut were monitored weekly during May – August in 2022 and 2023 and Gangs Bayou weekly during May – August 2023.

### Animals and samples

Approximately 160 black skimmer chicks were found dead on Gangs Bayou (n = 94) and Old Gulf Cut (n = 66). The chicks were 2−4 weeks-old, exhibiting first-winter plumage, and were in poor body condition. Six black skimmer chicks were included in the present study. From these, one chick was found dead and five chicks were found with lethargy, emaciation, and diarrhea and required euthanasia. Three chicks were euthanized using CO_2_ within 3 hours after collection and transportation to the research facility, and two chicks were euthanized by cervical dislocation in the field due to significant respiratory distress. All handling, sampling, and euthanasia protocols were approved by the Texas A&M Institutional Animal Care and Use Committee (IACUC 2021−0042), Texas Parks and Wildlife Department (permit SPR-0317–079) and U.S. Fish and Wildlife Service (MB66499C). The five euthanized individuals and one dead chick found in good preservation status were collected and transported in individual plastic bags with ice to the Department of Veterinary Pathobiology at Texas A&M University, where full necropsies were performed. Body condition was evaluated using a 1–5 keel score rating [28], considering the pectoral muscle mass in relation to the keel where 1 is emaciated (keel is sharply demarcated), 2 is poor (keel is prominent with small amount of muscle on each side), 3 is fair (keel is palpable with moderate amount of muscle on each side), 4 is good (keel palpable at the level of the pectoral muscles) and 5 is over conditioned (keel not palpable). Representative samples from the main organs including kidney, small intestine and cloaca were collected and preserved in 10% neutral buffered formalin for histopathology or frozen at −80°C for molecular analysis.

### Histopathology

The formalin fixed tissue sections were trimmed and placed in cassettes, embedded in paraffin, cut at 4 µm sections and stained with hematoxylin and eosin according to standard histologic procedures. The sections were evaluated through light microscopy. Periodic acid-Schiff staining was performed to highlight the coccidian stages in tissue.

### Molecular methods

Genomic DNA was extracted from frozen kidney samples using DNeasy^®^ Blood and Tissue DNA extraction kit (QIAGEN, Hilden, Germany) following the manufacturer’s protocol. Cloaca and small intestine samples were embedded as single tissues in different paraffin blocks. DNA from formalin-fixed and paraffin embedded sections of the cloaca and small intestine was extracted in duplicate using the QIAamp^®^ DNA FFPE Advanced Kit (QIAGEN, Hilden, Germany) following the manufacturer’s protocol. Amplification of a portion of the 18S rRNA gene was performed by polymerase chain reaction (PCR) in a total reaction volume of 25 µL containing 10 µM of each primer, 2x GoTaq^®^ Green Master Mix (Promega Corporation, Madison, Wisconsin, USA) and 2.5 µL of DNA template. The 18S rRNA portion was amplified using previously published primers EIMF (forward) 5’-ACCATGGTAATTCTATG-3’ and EIMR (reverse) 5’-CTCAAAGTAAAAGTTCC-3’ [5,29]. Thermocycling conditions consisted of an initial denaturation 95 °C for 2 minutes, followed by 35 cycles of 95 °C for 45 seconds, 45 °C for 1 minute, and 72 °C for 90 seconds, with a final extension at 72 °C for 5 min. PCR products were amplified on a 1.5% agarose gel in TAE buffer with GelRed^®^ (Biotium Inc., Hayward, California, USA) at 100 V for 30 minutes and visualized by UV light.

The obtained PCR products were purified using the E.Z.N.A^®^ Cycle Pure Kit (OMEGA Bio-Tek., Inc., Norcross, Georgia, USA) according to the manufacturer’s instructions and sequenced by the Sanger sequencing method. The sequence chromatograms were inspected for mixed peaks, low quality segments of the sequences were trimmed, and high quality consensus sequences were obtained. Sequence alignment and construction of the phylogenetic tree was performed using the Molecular Evolutionary Genetics Analysis (MEGA X) version 10.0.05 software and compared to nucleotide sequences available in the National Center of Biotechnology Information (NCBI) database [30]. The phylogenetic tree was inferred by using the Maximum Likelihood method and Jukes-Cantor (+G) model and bootstrap consensus was inferred from 1,000 replicates to determine branch reliability [30,31].

## Results

**Fig 1:**
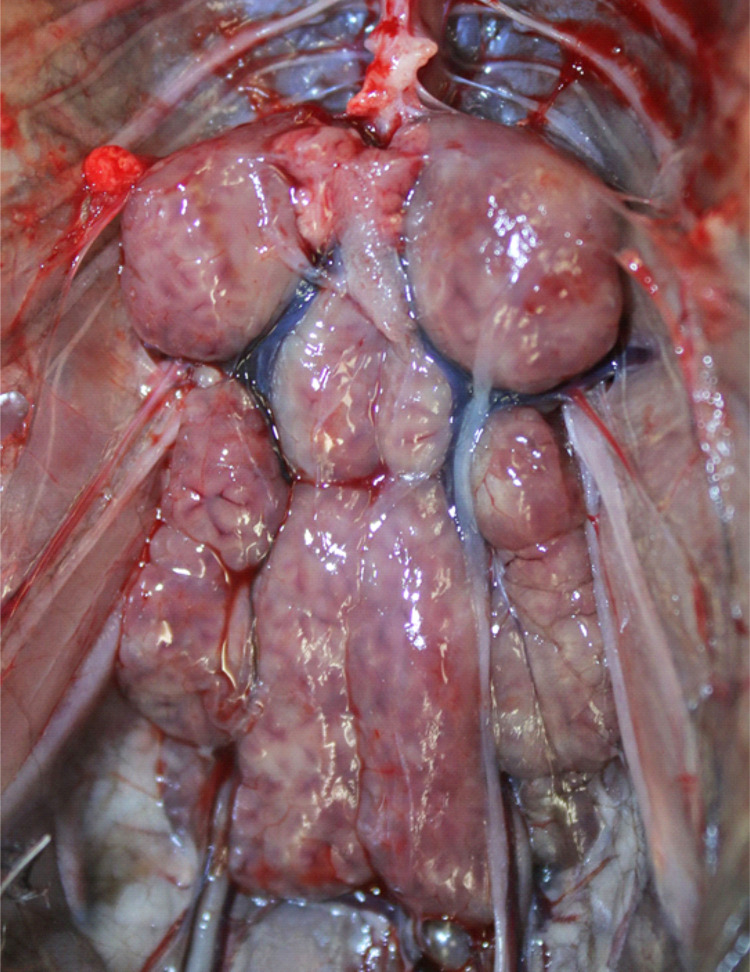
Kidney of a black skimmer, Case 5 (T23-N117). The kidneys are enlarged and have multifocal to coalescing pale tan areas.

**Fig 2:**
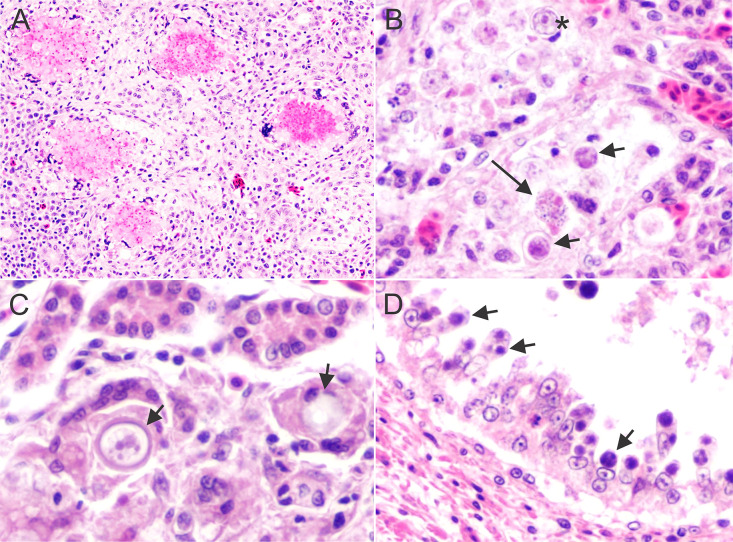
Histologic photomicrographs of kidney of black skimmers with coccidiosis. **A.** Case 1 (T22-N111). Renal cortex with multifocal areas of necrosis centered in degenerated oocysts, surrounded by macrophages and multinucleated cells. Lymphocytes, plasma cells and few heterophils are in the interstitium. Hematoxylin and eosin, 200x magnification; **B.** Case 5 (T23-N117). The medullary tubules are necrotic with cellular debris and multiple immature macrogamonts (short arrows), mature macrogamont (asterisk) and microgamonts (long arrow) in the lumen. Hematoxylin and eosin, 400x magnification; **C.** Case 5 (T23-N117). Oocysts (arrows) are in the cytoplasm of multinucleated giant cells. Hematoxylin and eosin, 600x magnification; **D.** Case 5 (T23-N117). The epithelium of medullary collecting tubules contains numerous intracytoplasmic gamonts. Hematoxylin and eosin, 600x magnification.

**Fig 3:**
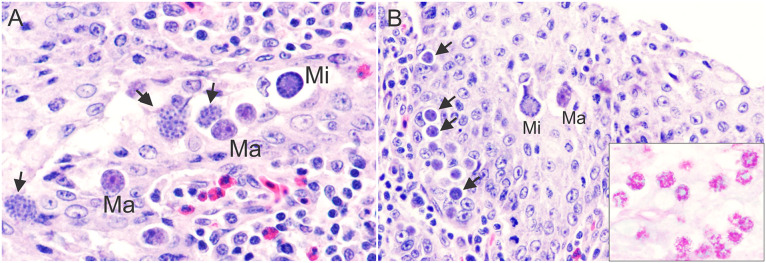
Histologic photomicrographs of the cloaca of a black skimmer with renal coccidiosis, Case 1 (T22-N111). **A.** The cloacal epithelium contains numerous intracytoplasmic and extracellular immature microgamonts (arrows), mature microgamonts (Mi) and immature macrogamonts (Ma). A few lymphocytes, plasma cells and heterophils are in the lamina propria. Hematoxylin and eosin, 600x magnification. **B.** Cloaca. Numerous intracytoplasmic gamonts are in the cloacal epithelium. Hematoxylin and eosin, 400x magnification; insert: PAS positive *Eimeria* sp. developmental stages. Periodic acid-Schiff, 600x magnification.

**Fig 4:**
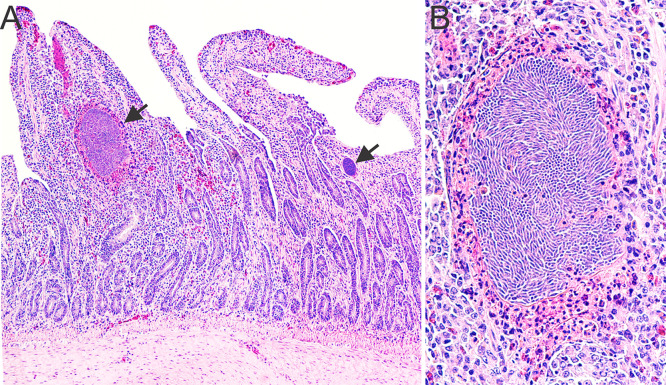
Histologic photomicrographs of the small intestine of a black skimmer with coccidiosis, Case 1 (T22-N111). **A.** The intestinal mucosa contains multiple schizonts (arrows). The mucosa is moderately infiltrated by eosinophils, lymphocytes and plasma cells. Hematoxylin and eosin, 40x magnification; **B.** The capsule of the schizont is ruptured, surrounded and infiltrated by a moderate numbers of eosinophils. Hematoxylin and eosin, 200x magnification.

The signalment, a summary of the pathologic findings, and molecular genetic results of the six black skimmer examined are presented in Table 1. Out of these six black skimmers, four (4/6, 67%) were in poor body condition. In three (3/6, 50%) cases, the kidneys were bilaterally enlarged, with multifocal to coalescing pale tan foci (Fig 1). On histology, multifocally the renal tubules, mostly distal convoluted tubules and collecting ducts, were dilated and lined by degenerated or necrotic epithelial cells that were often sloughed into the lumen and occasionally contained various developmental stages of coccidian organisms (immature gamonts, microgamonts, macrogamonts, and oocysts) in the cytoplasm (Fig 2). The immature gamonts were 3–6 µm in diameter and contained a round, deeply basophilic nucleus. The microgamonts were 11–14 µm in diameter, round to oval, and had numerous 1 µm basophilic nuclei (microgametes). The macrogamonts were 9–11 µm in diameter, round, and had a central amphophilic nucleus and peripheral eosinophilic granules. The oocysts were 13–17 µm in diameter, round to oval, had a 2–3 µm thick eosinophilic wall, and were filled with a 9–10 µm in diameter single central nucleus. A moderate number of macrophages, lymphocytes and heterophils, were within the tubules and infiltrated the adjacent interstitium. Intratubular multinucleated giant cells with intracytoplasmic gamonts and oocysts were observed in one case (1/6, 17%). Occasionally, within the cortex, medullary cone, and urinary space were multifocal aggregations of inflammatory cells centered on areas of necrosis with proteinaceous material and cellular debris, surrounded by a rim of multinucleated giant cells. In two cases (2/6, 33%) there was moderate, histiocytic, heterophilic, and lymphoplasmacytic cloacitis. Case 1 presented intralesional cloacal epithelial intracytoplasmic immature gamonts, microgamonts and macrogamonts (Fig 3). Schizonts were observed only in the lamina propria of the small intestine of case 1 (Fig 4). The schizonts were oval, with a wall, and measured 32x53 µm to 110x182 µm. Occasionally, the schizonts were ruptured, surrounded and infiltrated by a moderate number of eosinophils, with fewer heterophils and macrophages.

**Table 1:** Signalment, histopathologic and molecular results.

ID	Collection Date	Location	Sex	Weight	Age	Body condition	Euthanized	Pathologic findings	Positive PCR Result
Case 1, T22-N111	03-Aug-2022	Matagorda	Male	272 g	Juvenile	Good	Yes	Kidney: Moderate, multifocal, chronic, histiocytic, heterophilic, and lymphoplasmacytic tubulointerstitial nephritis with tubular necrosis, degeneration, atrophy, and loss, fibrosis, intratubular urates, and intratubular coccidian developmental stages. Cloaca: Moderate, multifocal, chronic, histiocytic, heterophilic, and lymphoplasmacytic cloacitis with intralesional coccidian developmental stages. Small intestine: Mild, multifocal, subacute, eosinophilic enteritis with intralesional schizonts.	Cloaca, kidney, small intestine
Case 2, T22-N112	03-Aug-2022	Matagorda	Male	280 g	Juvenile	Poor	No	Kidney: Mild, multifocal, chronic, histiocytic, heterophilic, and lymphoplasmacytic tubulointerstitial nephritis with tubular degeneration, necrosis and intratubular urates. Cloaca: Mild, multifocal, chronic, histiocytic, heterophilic, and lymphoplasmacytic cloacitis.	Kidney
Case 3, T22-N114	18-Aug-2022	Matagorda	Female	226 g	Juvenile	Poor	Yes	Kidney: Severe, multifocal, multifocal, chronic, histiocytic, heterophilic, and lymphoplasmacytic tubulointerstitial nephritis with tubular necrosis, degeneration, atrophy, and loss, fibrosis, intratubular urates, and intratubular coccidian developmental stages.	Kidney
Case 4, T22-N115	18-Aug-2022	Matagorda	Male	302 g	Juvenile	Poor	Yes	Kidney: Mild, multifocal, chronic, lymphoplasmacytic, histiocytic, and heterophilic tubulointerstitial nephritis with intralesional coccidian developmental stages.	Kidney
Case 5, T23-N117	28-Jul-2023	Gangs Bayou, Galveston	Male	170 g	Juvenile	Poor	Yes	Kidney: Moderate, multifocal, chronic histiocytic, heterophilic, and lymphoplasmacytic tubulointerstitial nephritis with multinucleated giant cells, necrosis and intralesional coccidian developmental stages	Kidney
Case 6, T23-N118	28-Jul-2023	Gangs Bayou, Galveston	Male	203 g	Juvenile	Fair	Yes	Kidney: Mild, multifocal, chronic, lymphoplasmacytic tubulointerstitial nephritis with focal necrosis.	Kidney

Signalment and histopathologic findings in Black skimmers (*Rynchops niger niger*) from West Galveston and East Matagorda Bay, Texas, USA.

**Fig 5:**
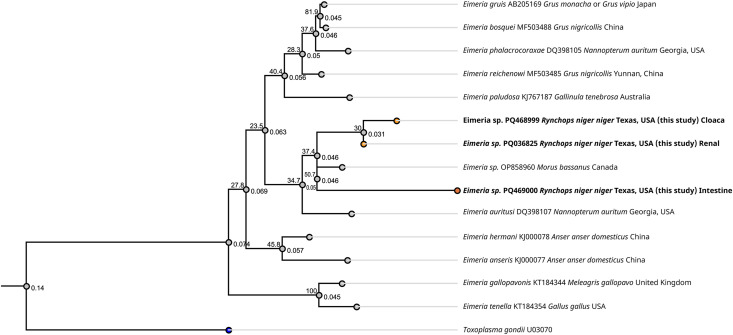
Phylogenetic tree. Consensus phylogenetic relationships of the renal, cloacal and intestinal *Eimeria* lineage from this study based on a fragment of the 18S rRNA gene. Percentage of 1,000 bootstrap samples that supported clades on branches using the maximum likelihood method. Jukes-Cantor + G was used as the best substitution model. *Toxoplasma gondii* is used as an outgroup.

The molecular analysis of five out of six renal samples and both the cloacal and intestinal samples produced amplicons of approximately 600 bp (S1 Fig). After trimming out low quality readings, fragments of 490 bp, 372 bp and 374 of the 18S rRNA gene were obtained from the kidney, cloaca and intestinal samples. The renal samples were 100% identical to one another and a consensus sequence was submitted to GenBank under accession PQ036825. The cloacal sample was 99.5% similar to the renal samples, differing from 2 nucleotides, and was submitted to GenBank under accession PQ468999. The intestinal sample had 96.25% identity to the renal sample, differing from 13 nucleotides considering the alignment of 347 bp, and was submitted to GenBank under accession PQ469000. The sequences were analyzed using the BLASTn tool to determine their similarity to published sequences in the NCBI nr/nt database. As of June 2nd, 2025, the consensus sequence from the kidney sample had 96.25% identity with *Eimeria reichenowi*, identified in red-crowned cranes (*Grus japonensis*) in Japan (accession numbers AB544352, AB544354 and AB544350) [32]. The sequence from the small intestine of the black skimmer had 97.04% similarity to a novel *Eimeria* sp. identified in Northern gannets (*Morus bassanus*), in Canada (accession number OP858960) [33]. A phylogenic tree of the renal, cloacal, and intestinal sequences compared with closely related *Eimeria* spp. was constructed using *Toxoplasma gondii* as an out-group (Fig 5).

The cloacal, renal, and intestinal *Eimeria* from the black skimmers formed a separate clade, together with the *Eimeria* sp. found in Northern gannets. While the renal *Eimeria* lineage detected in this study likely represents a novel, previously uncharacterized lineage, a formal taxonomic description of the lineage or species would require further studies based on the morphology of sporulated oocysts.

## Discussion

In the present study we describe the pathologic findings and molecular analysis of renal coccidiosis caused by *Eimeria* infection in six black skimmers that died during an outbreak of mortality in East Matagorda Bay and West Galveston, Texas, USA. Additionally, two of these skimmers had evidence of *Eimeria* developmental stages in the cloaca and one had intestinal coccidiosis. To the best of our knowledge, this is the first report of *Eimeria* infection in a *Rynchops* species.

Malnutrition, other concurrent diseases, and immunosuppression could have predisposed the renal coccidiosis outbreak in black skimmers. Generally, the incidence of renal coccidiosis is higher in juveniles than adults [5,8]. In the present study, morbidity and mortality were observed in young black skimmers with renal coccidiosis, being histologically and molecularly confirmed in all animals examined. Adult black skimmers were not included in our study, and it is possible that the adults are subclinically infected. Further studies, including the survey of *Eimeria* on tissues and excreta from a larger number of animals, are necessary to determine the prevalence of *Eimeria* sp. infection in this population and the epidemiologic role of adult black skimmers.

*Eimeria auritusi* has also been detected in apparently clinically healthy double-crested cormorants [5]; however, macroscopic lesions are not always evident in avian renal coccidiosis [8]. In the present study, bilateral renomegaly with multifocal discrete to coalescing pale foci, which are consistent with the areas of inflammation and necrosis, was observed histologically. Similar lesions have been reported in cases of renal coccidiosis in waterfowl [6,34]. The histologic lesions observed in black skimmers were also consistent with previously reported cases of renal coccidiosis in other avian hosts [8]. Distal convoluted tubules and collecting ducts were the most affected with tubular necrosis and predominant histiocytic inflammation, associated to various developmental stages in the cytoplasm of epithelial cells.

*Eimeria* species are generally host-specific and therefore often considered monoxenous parasites [35]; however, in many cases an *Eimeria* species can affect multiple closely related species. Eleven *Eimeria* species have been described in the family Laridae: *E. argentati, E. lari, E. mellumi, E. wobeseri* and *E. goelandi* in herring gulls (*Larus argentatus*) [36,37], *E. creutzi* and *E. renicola* in black-headed gull (*Chroicocephalus ridibundus*) [38], and *E. undata* in *L. argentatus,* tufted puffin (*Lunda cirrhata*)*,* common murre (*Uria aalge aalge*) [36], *E. rissae* in black-legged kittiwake (*Rissa* *tridactyla*) [39], *E. meservei* in (*Sterna forsteri*) [40], and *E. atricillae* in laughing gull (*Leucophaeus atricilla*) [41]. In most cases, *Eimeria* developmental stages were found infecting the gastrointestinal tract or were detected in fecal samples, with only three species, *E. renicola*, *E. goelandi*, and *E. wobeseri* found infecting the urinary tract and causing renal coccidiosis [37,38]. Direct comparison of the *Eimeria* found in our study is limited to those studies that include DNA sequencing. A limitation of our study is that we could not perform culture and include the morphologic description of sporulated oocyts.

The *Eimeria* lineage found in the kidneys and cloaca of black skimmers in the present study is relatively closely related to *Eimeria reichenowi*, found in a variety of crane species, associated with disseminated visceral coccidiosis, resulting in hepatitis, bronchopneumonia, myocarditis, splenitis, and enteritis [17,42,43]. However, the nucleotide differences between *E. reichenowi* and the *Eimeria* sp. detected in the kidneys and cloaca of black skimmers indicate that they are distinct lineages. Although the renal and cloacal samples differed at two bases, it has been previously demonstrated that apicomplexan parasites can produce divergent copies of the 18S rRNA from the same *Eimeria* oocyst line indicating the *Eimeria* found in both the kidney and cloaca are most likely the same species [31].

*Eimeria auritusi*, another species of *Eimeria* closely related to the lineage found in the kidneys and cloaca of black skimmers, have caused fatal renal coccidiosis outbreaks of double-crested cormorants (*Nannopterum auritum,* formerly *Phalacrocorax auritus*) from Chatham County, Georgia, USA [15]. Similarly to our study, the renal lesions caused by *E. auritusi* infection were associated with significant morbidity and likely contributed to mortality [15]. It is noteworthy to mention that there are no available sequences for the three *Eimeria* species associated with renal coccidiosis in other Laridae hosts, precluding a direct comparison.

The *Eimeria* lineage found in the intestine of a black skimmer in the present study is closely related to an uncharacterized *Eimeria* species identified in Northern Gannets along the coasts of New Brunswick, Nova Scotia, and Prince Edward Island in Canada [33]. In that case, 220 birds were evaluated over 27 years and the prevalence of renal coccidiosis was 35.5%, including adults, immature and hatch-year birds without significant clinical disease [33]. In the present case, intestinal coccidiosis was found in only one individual. The associated lesions were focal inflammation in response to the rupture of the schizonts. Other coccidian developmental stages were not appreciated in the sections of intestine examined. The significance of the intestinal coccidian infection in the only bird affected in our study is likely lower than the renal coccidiosis. A similar case was observed in little penguins (*Eudyptula minor*) during an event of increased mortality associated with poor body condition and heavy parasitic burden, where 64.5% (20/31) of the birds evaluated had chronic interstitial nephritis with renal coccidiosis and only one had evidence of schizonts in the small intestine [11].

In conclusion, young black skimmers seem to be susceptible to renal coccidiosis caused by a novel *Eimeria* lineage based on molecular analysis. Additionally, another uncharacterized *Eimeria* lineage was found in the intestine of a black skimmer. Further studies based on the morphology of sporulated oocysts would be necessary to formally describe and characterize these new *Eimeria* lineage. The significance of renal coccidiosis in black skimmers is unknown. Although only six birds were available for the present study, the high prevalence of renal coccidiosis and significant renal dysfunction likely contributed to mortality of nestlings and to population decline of black skimmers in the Texas Gulf coast. Environmental stressors and other concurrent diseases could have also contributed to the mortality outbreak; complete pathologic investigation of a higher number of individuals would be valuable to elucidate potential factors affecting the health of this black skimmer population.

## Supporting information

S1 Fig Gel electrophoresis results of black skimmers with coccidiosis. Positive samples have the expected 600 bp band. Negative sample have no band. A) Fresh kidney. 1–6) Cases 1–6, 7) DNA size marker. B) Formalin-fixed paraffin embedded tissues from case 1 (T22-N111). 1–2) Cloaca, 3–5) Kidney, 4) No template control, 5–7) Intestine, 6) DNA size marker.
(TIF)
