# Correction: Renal coccidiosis in black skimmers in North America reveals an uncharacterized *Eimeria* lineage (Apicomplexa: Eimeriidae)

**DOI:** 10.1371/journal.pone.0359440

**Published:** 2026-09-28

**Authors:** Kátia R. Groch, Tiana L. Sanders, Elena M. I. Duran, Susan A. Heath, Dallas Clontz, Jacquelyn K. Grace, Raquel R. Rech, Guilherme G. Verocai, Laura K. Bryan

Susan A. Heath should be included in the author byline instead of the Acknowledgments. Susan A. Heath should be listed as the fourth author and affiliated with the Gulf Coast Bird Observatory, 299 Hwy 332 West, Lake Jackson, Texas 77566, United States of America. The contributions of this author are as follows: Conceptualization, Data Curation, Funding Acquisition, Investigation, Methodology, Project Administration, Resources, Supervision, Writing – Review & Editing.

The correct citation is: Groch KR, Sanders TL, Duran EMI, Heath SA, Clontz D, Grace JK, et al. (2026) Renal coccidiosis in black skimmers in North America reveals an uncharacterized *Eimeria* lineage (Apicomplexa: Eimeriidae). PLoS One 21(4): e0345982. https://doi.org/10.1371/journal.pone.0345982

There are errors in the Funding statement. The correct Funding statement is as follows: This study was financially supported by Texas A&M University in the form of a Grants-In-Aid of Graduate Research Program grant awarded to ED. This study received additional financial support from the Lewis and Clark Foundation in the form of a Lewis and Clark Fund for Exploration and Field Research grant awarded to ED. Finally, the study was supported by the Gulf Coast Bird Observatory which provided financial and in-kind support.
