# Supplementary figures and images for: Renal coccidiosis in black skimmers in North America reveals an uncharacterized Eimeria lineage (Apicomplexa: Eimeriidae)
Source: PLoS One. 2026 Apr 16;21(4):e0345982. doi: 10.1371/journal.pone.0345982 (PMC13086328; doi:10.1371/journal.pone.0345982)

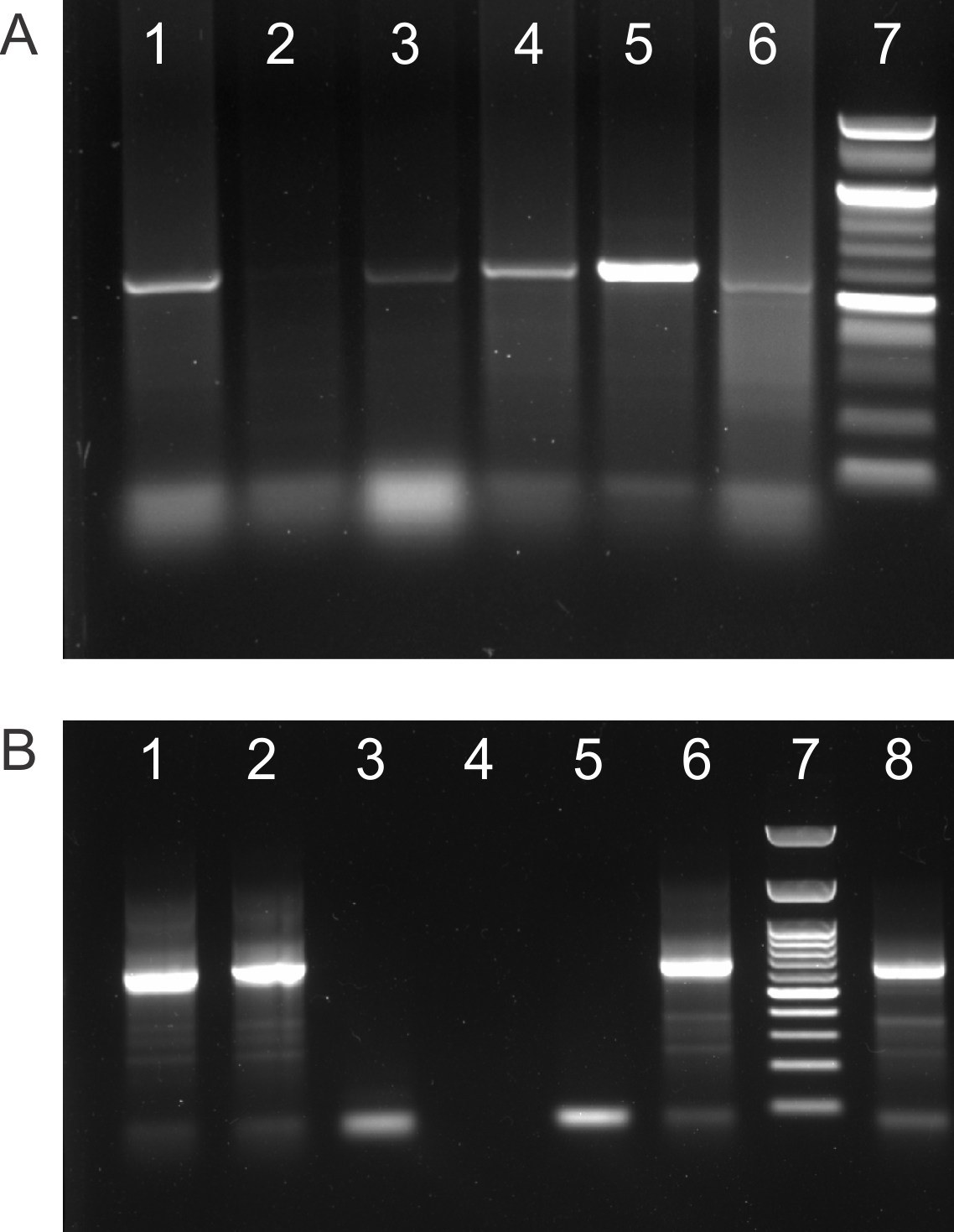

Supplement: S1 Fig — Positive samples have the expected 600 bp band. Negative sample have no band. A) Fresh kidney. 1–6) Cases 1–6, 7) DNA size marker. B) Formalin-fixed paraffin embedded tissues from case 1 (T22-N111). 1–2) Cloaca, 3–5) Kidney, 4) No template control, 5–7) Intestine, 6) DNA size marker. (TIF) [file pone.0345982.s001.tif]
